# Porokeratosis ptychotropica mit mykotischer Superinfektion bei einem 68-jährigen Mann

**DOI:** 10.1007/s00105-025-05594-x

**Published:** 2025-10-10

**Authors:** Jana Roßner, Alexander Enk, Ferdinand Toberer

**Affiliations:** Universitäts-Hautklinik Heidelberg, Im Neuenheimer Feld 440, 69120 Heidelberg, Deutschland

## Abstract

Die Porokeratosis ptychotropica stellt eine sehr seltene Unterform der Porokeratosen, einer heterogenen Gruppe erblicher Verhornungsstörungen, dar. Es kommt zu verrukösen, großflächigen, hyperkeratotischen Plaques in der Perigenital‑, Gluteal- und Inguinalregion. Histologisch weisen die Porokeratosen eine pathognomonische Veränderung auf, die sog. kornoide Lamelle. Die Behandlungsergebnisse sind meist enttäuschend und mit hohen Rezidivraten verbunden.

## Abstract (en)

Porokeratosis ptychotropica is a very rare subtype of porokeratosis, which is a heterogeneous group of inherited keratinization disorders. It is associated with verrucous, extensive hyperkeratotic plaques in the perigenital, gluteal, and inguinal regions. Histologically, porokeratosis exhibits the pathognomonic hallmark known as cornoid lamellae. Treatment results are generally disappointing and recurrence rates are high.

## Anamnese

Wir berichten über den Fall eines 68-jährigen Patienten, der sich mit einer seit ca. 40 Jahren bestehenden Hautveränderung am Gesäß vorstellte.

Der Patient berichtete, dass sich die Hautveränderung über die Jahre langsam nach peripher ausgebreitet hätte. Er klagte über starken Juckreiz und ein brennendes Gefühl beim Sitzen im Bereich der Läsion. Der Patient hatte keine Vorerkrankungen und keine bekannten Allergien. Die Familienanamnese bezüglich Hauterkrankungen war negativ. Auslandsaufenthalte vor Auftreten der Veränderung sowie der Kontakt zu Tieren wurden verneint.

## Untersuchung

**Abb. 1:**
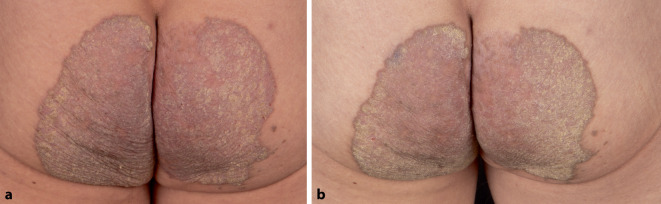
**a** Klinisches Bild: an beiden Gesäßhälften nahezu symmetrische, livid-erythematöse, scharf begrenzte, verruköse Plaques mit Randbetonung und gelblicher Schuppung sowie wenigen peripheren Satellitenherden. **b** Klinisches Bild nach abgeschlossener antimykotischer Therapie: deutliche Regredienz der Hyperkeratosen

Bei der körperlichen Untersuchung fanden sich an beiden Gesäßhälften nahezu symmetrische, livid-erythematöse, scharf begrenzte, verruköse Plaques mit Randbetonung und gelblicher Schuppung sowie wenigen peripheren Satellitenherden (Abb. 1a).

Das restliche Integument, die Mund- und Genitalschleimhaut sowie die Kopfhaut zeigten keine Auffälligkeiten.

## Diagnostik

Die mykologische Multiplex-real-time-PCR(Polymerasekettenreaktion)-Untersuchung der Schuppen von gluteal zeigte sich positiv für *Trichophyton rubrum*.

**Abb. 2:**
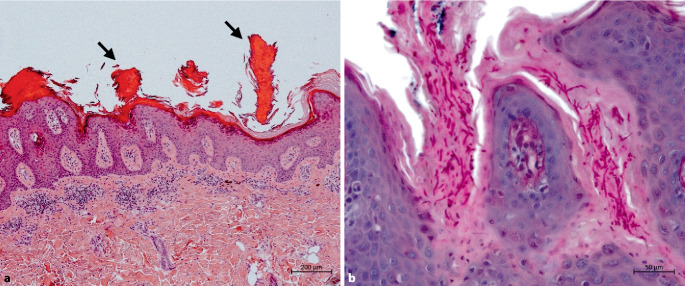
**a** Hautbiopsat von gluteal: zahlreiche Säulen bzw. schlotartig angeordnete Hyperparakeratosestreifen (*schwarze Pfeile*), die in grübchenartigen Vertiefungen der Epidermis verankert sind, die sog. kornoiden Lamellen (Hämatoxylin-Eosin-Färbung, Originalvergrößerung 50:1, Maßstabbalken = 200 µm). **b** In der Alcian-PAS(Periodic-Acid-Schiff)-Färbung zeigen sich zahlreiche Pilzhyphen, betont im Bereich der kornoiden Lamellen

Wir führten eine Stanzbiopsie vom Randbereich der Läsion durch, wobei sich histologisch zahlreiche sog. kornoide Lamellen (Hyperparakeratosesäulen, die in grübchenartigen Vertiefungen der Epidermis verankert sind) nachweisen ließen (Abb. 2a). In der Alcian-PAS(Periodic-Acid-Schiff)-Färbung imponierten zahlreiche Pilzhyphen in der Hornschicht (Abb. 2b).

## Diagnose

- Porokeratosis ptychotropica mit mykotischer Superinfektion

## Therapie und Verlauf

Es wurden eine orale antimykotische Therapie mit Terbinafin 250 mg pro Tag über 4 Wochen sowie eine begleitende Lokaltherapie mit Ciclopirox-haltiger Creme 1‑mal täglich im Wechsel mit Calcipotriol-Creme 1‑mal täglich eingeleitet. Darunter kam es bei dem Patienten zu einer Regredienz der Hyperkeratosen (Abb. 1b). Die anschließend erfolgte mykologische Multiplex-PCR zeigte sich negativ. Das Ansprechen auf eine orale Retinoidtherapie bleibt abzuwarten.

## Diskussion

Die Porokeratosen umfassen eine heterogene Gruppe seltener, autosomal-dominant erblicher Verhornungsstörungen. Sie sind durch eine charakteristische gemeinsame Morphologie gekennzeichnet. Typisch sind nummuläre, zentral oft atrophe Papeln bis Plaques mit wallartigem Rand [1]. Es wird eine langsame, zentrifugale Ausbreitung der Hautveränderungen ohne Spontanheilungstendenz beschrieben. Histologisch weisen die Porokeratosen eine pathognomonische Veränderung auf, die als kornoide Lamelle bezeichnet wird. Die kornoide Lamelle zeigt eine säulenartige Parakeratose über einer fokalen Hypogranulose, häufig unterlegt von Dyskeratosen im Stratum spinosum und granulosum. Dies reflektiert eine gestörte terminale Keratinozytendifferenzierung [2]. Es sind mehrere Hauptformen der Porokeratosen bekannt, hierunter die klassische Porokeratosis Mibelli, die Porokeratosis palmaris, plantaris et disseminata und die Gruppe der disseminierten superfiziellen aktinischen Porokeratosen [1].

Die Porokeratosis ptychotropica (PP) gilt als eine der seltensten klinischen Varianten der Porokeratose und ist bislang nur in wenigen Fallberichten und kleinen Fallserien beschrieben worden [3]. Prädilektionsstellen sind die Perigenital‑, Gluteal- und Inguinalregion. Klinisch imponieren bei dieser Unterform großflächige, verruköse, hyperkeratotische Plaques mit ausgeprägtem Juckreiz. Betroffen sind v. a. Männer mittleren Alters. Eine Abgrenzung zu relevanten Differenzialdiagnosen wie Psoriasis, Lichen ruber oder chronischem Ekzem stellt häufig eine klinische Herausforderung dar [3, 4].

Der Entstehung von Porokeratosen liegt eine klonale Expansion atypischer Keratinozyten zugrunde. Molekulargenetische Studien der letzten Jahre haben gezeigt, dass bei vielen Formen Mutationen im Mevalonat-Stoffwechselweg nachweisbar sind. Diese führen zu einer gestörten Cholesterin- und Isoprenoid-Biosynthese und damit zu einer fehlerhaften terminalen Differenzierung der Keratinozyten. Das pathophysiologische Modell des „Two-Hit“-Prinzips wird zunehmend unterstützt. Demnach schafft eine Keimbahnmutation eine genetische Prädisposition, während ein somatisches Ereignis (z. B. UV-induzierte DNA[Desoxyribonukleinsäure]-Schädigung) zur klinischen Manifestation führt [5, 6].

Neben genetischen Faktoren spielen auch exogene und endogene Kofaktoren eine wichtige Rolle. Hierzu zählen Immunsuppression (u. a. nach Organtransplantation), UV-Exposition, bestimmte Medikamente (z. B. Hydroxyurea, Thiaziddiuretika, TNF[Tumornekrosefaktor]-α-Inhibitoren) sowie virale Infektionen (z. B. HCV [Hepatitis-C-Virus], HPV [humane Papillomviren], HSV [Herpes-simplex-Virus]). Auch systemische Erkrankungen wie rheumatologische Autoimmunerkrankungen, hämatologische Neoplasien und solide Tumoren sind mit einem erhöhten Risiko für Porokeratosen assoziiert. Diese Faktoren scheinen die klonale Expansion mutierter Keratinozyten zu fördern und damit die Krankheitsentstehung zu begünstigen [2].

Für nahezu alle Typen der Porokeratosen sind Fälle der malignen Entartung beschrieben. Vor allem bei großflächigen Herden mit langjährigem Bestehen konnte der Übergang in einen Morbus Bowen, ein Plattenepithelkarzinom oder ein Basalzellkarzinom beobachtet werden. Der Prozess der malignen Entartung bei der PP ist sehr selten, in der Literatur findet sich bisher nur ein einziger Fall [7].

Die Behandlungsergebnisse der Porokeratosen sind in den meisten Fällen enttäuschend, geprägt von schlechtem Ansprechen und hohen Rezidivraten.

Es gibt keine internationalen Therapieleitlinien. Die Therapieempfehlungen basieren auf Fallberichten/-serien, und die Entscheidung richtet sich nach der Variante und der Ausbreitung. Ein Vorgehen nach dem „watch-and-wait approach“ unter angemessenem Sonnenschutz und regelmäßiger, langfristiger Nachsorge ist eine gute Option für asymptomatische Patienten [2]. Die Therapieoptionen bei der PP reichen von topischen Steroiden und Immunmodulatoren über topische sowie orale Retinoide, PDT(photodynamische Therapie)-Behandlung, CO_2_(Kohlenstoffdioxid)-Laser, Dermabrasio bis hin zur Exzision [4]. Ein aktueller Fallbericht beschreibt eine erfolgreiche Therapie der PP mit dem JAK(Januskinase)-1-Inhibitor Abrocitinib [8]. In jüngster Vergangenheit haben sich topisches Simvastatin sowie die Kombination aus topischem Cholesterin und Simvastatin als effektive, lang anhaltende und gut verträgliche Therapieoption bei Patienten mit PP erwiesen [9]. Eine Kombination aus pathogeneseorientierter Lokaltherapie mit ablativen Maßnahmen mittels Dermabrasio bei ausgedehnten hyperkeratotischen Plaques wurden an unserem Zentrum klinisch erfolgreich eingesetzt.

## Fazit für die Praxis

- Der Fall des hier vorgestellten Patienten verdeutlicht, dass bei langjährig bestehenden, therapierefraktären Psoriasis-ähnlichen Plaques in der Glutealregion auch an seltene Erkrankungen wie die Porokeratosis ptychotropica (PP) gedacht werden sollte.
- Die Gewinnung einer Probebiopsie vom Randbereich der Hautveränderung kann durch die spezifische Histologie mit der sog. kornoiden Lamelle entscheidend zur Diagnosestellung beitragen.
- Die Behandlung verläuft oft unbefriedigend. Neue pathogeneseorientierte Therapieansätze bieten womöglich vielversprechende Perspektiven und lassen auf positive Entwicklungen hoffen.
- Eine mykotische Superinfektion der PP ist extrem selten, sollte jedoch bei entsprechendem Verdacht abgeklärt und behandelt werden.
